# Pediatric puzzle: Large ovarian dermoid cyst and markedly elevated CA 19-9 in an 8-year-old

**DOI:** 10.5339/qmj.2024.47

**Published:** 2024-09-16

**Authors:** Muhamed Ahmed Abdelmoaty, Wael Soliman Taha

**Affiliations:** 1 Department of Obstetrics and Gynecology, Faculty of Medicine, Al-Azhar University, Cairo, Egypt *Email: MuhamedAhmed.216@azhar.edu.eg

**Keywords:** Dermoid cyst, ovarian, CA-19-9 antigen, teratoma, cystic, ultrasonography

## Abstract

**Background:**

Mature cystic teratomas, also known as dermoid cysts, are the most prevalent form of ovarian germ cell tumors. While they typically manifest in women of reproductive age, they can also occur in pediatric patients. These tumors are generally benign and comprise a diverse array of tissue types. However, large lesions, particularly those exceeding 10 cm in diameter, are infrequent and can present diagnostic and therapeutic challenges. Notably, elevated tumor markers, such as cancer antigen 19-9 (CA 19-9), are not commonly associated with mature cystic teratomas, rendering this case particularly unusual.

**Case presentation:**

The clinical case involved an 8-year-old female patient who presented with an exceptionally large ovarian teratoma, measuring 13 × 12 cm. While the prepubertal presentation of such tumors is not uncommon, the remarkable size of the lesion was an extraordinary occurrence. Preoperative evaluation revealed markedly elevated levels of CA 19-9, a tumor marker, at 297 U/mL—an atypical finding for mature cystic teratomas. Imaging studies identified a complex cystic adnexal mass, indicative of a teratoma. Consequently, a laparotomy was performed, revealing an intact, benign lesion that was successfully resected via cystectomy, with preservation of the ovary. Histopathological examination confirmed the diagnosis of a mature cystic teratoma, without any evidence of malignant transformation. Notably, following the surgical intervention, the elevated CA 19-9 levels normalized, suggesting a potential association between the teratoma and the abnormal tumor marker levels.

**Discussion:**

This report delineates the surgical management and clinical course of an exceptionally large ovarian teratoma in a pediatric patient with abnormal preoperative tumor markers. Despite atypical features, the excellent prognosis following fertility-sparing resection underscores the significance of conservative treatment in young females.

**Conclusion:**

This case highlights the occurrence of a large mature cystic teratoma with elevated CA-19-9 in a pediatric patient with no complications such as torsion, rupture, or malignancy. The elevation in CA-19-9 likely relates directly to the teratoma itself. A conservative, fertility-sparing surgical approach proved effective, emphasizing the importance of careful preoperative evaluation and management in similar cases.

## 1. Introduction

Mature cystic teratomas, known as dermoid cysts, are germ cell tumors originating from ovarian tissue that contain developmentally mature tissue from all three germ cell layers.^1^ They are the most frequent subtype of ovarian germ cell tumors, accounting for approximately 11% of all ovarian neoplasms and 69% of all germ cell tumors. The reported incidence of mature cystic teratomas varies widely, ranging from approximately 1.2 to 14.2 cases per 100,000 individuals annually.^2^

The typical presentation of mature cystic teratomas is in patients during their reproductive years, with reported age ranges from 13 to 76 and a median age in the mid-30s.^3^ While most mature cystic teratomas are benign, the literature estimates a 0.5–3% frequency of malignant transformation, which appears to be greater in older women.^4^ Squamous cell carcinoma is the most common histologic type among individuals undergoing malignant transformation.^5^

The clinical presentation of ovarian dermoid cysts is highly variable. Approximately 20% of cases are asymptomatic, often discovered incidentally during imaging studies or surgical procedures.^6,7^ Symptomatic individuals may present with chronic pelvic pain, increased pelvic pressure, or acute abdominal pain resulting from complications such as ovarian torsion or rupture.^7-9^

The most commonly used imaging approach for confirming ovarian dermoid cysts is ultrasound (US), which is accurate enough to provide a diagnosis that can be verified by postoperative histology.^10^ The presence of a dermoid plug or Rokitansky nodule on imaging is regarded as strong radiographic evidence supporting a teratoma diagnosis.^11^ Dermoid cysts can be diagnosed with computed tomography or Magnetic Resonance Imaging (MRI), both of which are more sensitive to fat than US.^12^

On gross examination, ovarian dermoid cysts typically appear as unilocular cysts containing Rokitansky nodules, which include hair, teeth, and other tissues (Figure 3).^13^ Histologically, these tumors are characterized by well-differentiated tissues originating from all three germ layers. Ectodermal tissues and sebaceous material are almost universally present. The cyst cavity is filled with sebaceous material, and the lining is composed of squamous epithelium. Mesodermal tissues, such as fat, bone, cartilage, and muscle, are present in over 90% of cases, while endodermal tissues are encountered less frequently.^2^

Immunohistochemistry (IHC) is a highly sensitive technique used to identify tissue antigens with specific antibodies visible under a microscope. It is crucial in various research and pathological applications, including histogenetic diagnosis, neoplasia subtyping, identifying primary sites of malignant tumors, assessing prognostic factors, and differentiating between benign and malignant cell proliferations.^14^ IHC is often employed in cases of malignant transformation in mature cystic teratomas to detect specific antigens and assist in management.^15^

Cancer antigen 19-9 (CA 19-9), also known as sialylated Lewis (a) antigen, is a tumor marker found in cell membranes that undergoes changes throughout malignant transformation. CA 19-9 is most typically high in pancreatic, biliary, colonic, esophageal, and hepatic cancers, although it can also be elevated in some benign diseases.^16^ CA 19-9 has recently been found as a possible diagnostic marker for ovarian mature cystic teratomas, despite the fact that it is not typically increased in most instances.^17^ Serum CA 19-9 levels in mature cystic teratomas are infrequently documented in the literature,^18^ especially in younger individuals, and are generally linked with complications such as torsion.^19^

To the best of the authors’ knowledge, this case involves a prepubertal female with an ovarian mature cystic teratoma who had the youngest age at presentation and a high CA 19-9 level. Institutional protocol was followed, and formal parental approval was obtained before publishing this case report with images.

## 2. Case Presentation

An 8-year-old female presented to our clinic with a history of chronic abdominal pain and distension. Physical examination revealed a mobile pelvic-abdominal mass extending beyond the umbilicus. Ultrasonography demonstrated a 13 × 12 cm complex cystic lesion in the pelvis with a peripheral solid component and preserved peripheral ovarian blood flow on Doppler. MRI confirmed these findings, with the cystic portion displaying low T1 and high T2/STIR signal intensity, while the solid area showed high T1 and low T2/STIR signal with foci of susceptibility artifact concerning for calcifications.

Laboratory evaluation revealed normal levels of beta-human chorionic gonadotropin, alpha-fetoprotein, and carcinoembryonic antigen at <2, 1.85, and 1.88 ng/mL, respectively. Cancer antigen 125 was normal at 12.2 U/mL. However, CA 19-9 was significantly elevated at 297 U/mL (normal range 2.5–33.5 U/mL). Gastrointestinal malignancies were ruled out on imaging.

**Figure 1.**
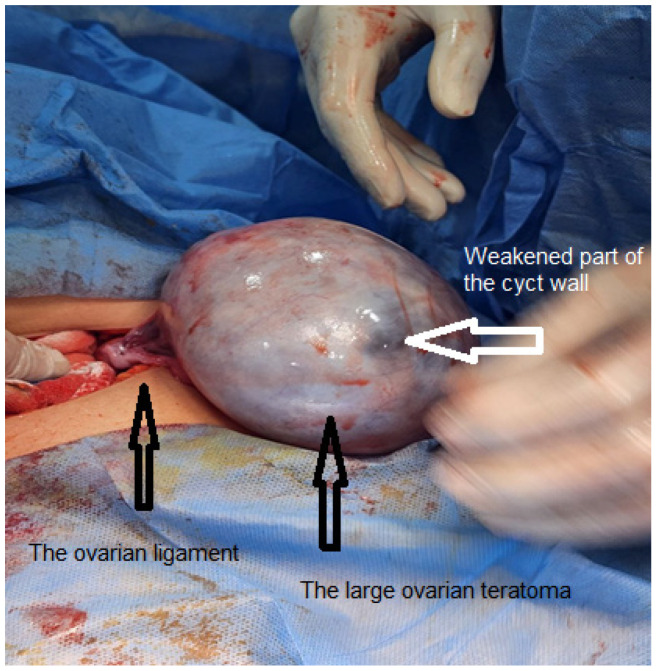
Ovarian cyst before the excision. Note the weak wall of the cyst in some areas.

**Figure 2.**
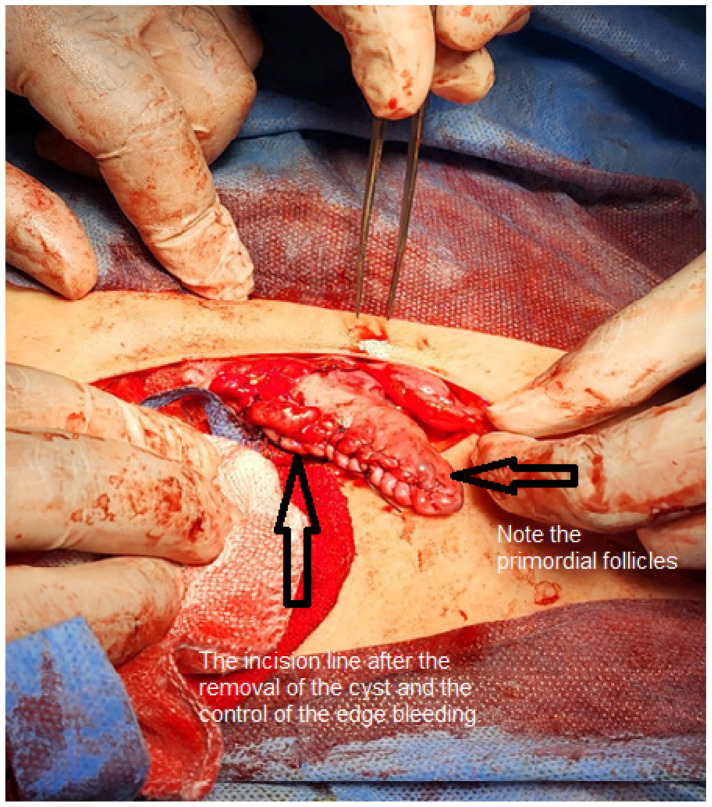
Reconstruction of the ovary after complete excision of the cyst.

**Figure 3.**
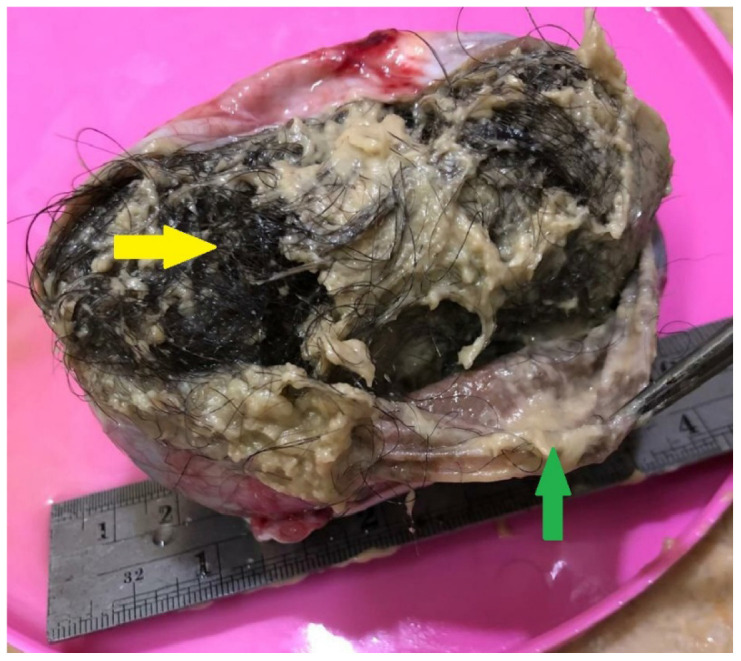
Cut section of the cyst. Not the hair follicles (yellow arrow) and the sebaceous material (green arrow).

The patient underwent surgical excision of the cyst 2 weeks after initial presentation to our clinic via a Pfannenstiel skin incision and longitudinal rectus fascia opening to facilitate removal (Figure 1). Cystectomy was performed without cyst wall rupture, with ovarian preservation after cortical reconstruction (Figure 2). The remaining pelvic organs and peritoneum appeared normal on visual inspection. Histopathological examination confirmed the diagnosis of a benign mature cystic teratoma. The patient was admitted to our department for a total of 12 days, consisting of 7 days prior to the surgery and 5 days post-surgery. The abnormal preoperative CA 19-9 level normalized within 4 weeks postoperatively.

## 3. Discussion

**Figure 4.**
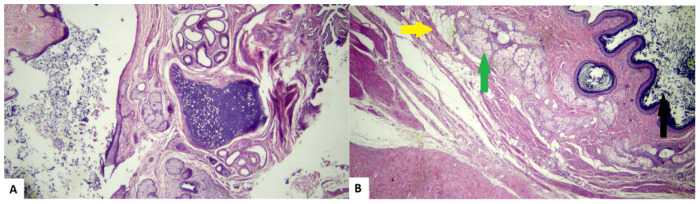
Histopathological assessment of the cyst. (A) Cyst wall lined by stratifies squamous epithelium with skin appendages with loose keratin flakes in the lumen. (B) Adipose tissue (yellow arrow), sebaceous glands (green arrow), and hair follicles.

Mature cystic teratomas (MCTs), commonly known as dermoid cysts, are the most prevalent benign ovarian neoplasms in women of reproductive age and adolescents. These masses account for approximately 70% of benign ovarian masses before menopause and a substantial 20% thereafter.^20,21^ Histologically, MCTs consist of mature tissues derived from the three germ cell layers: ectoderm, mesoderm, and endoderm (Figure 4). As a result, they contain a wide variety of tissue types, including skin, hair, teeth, fat, muscle, thyroid, and even neural elements. The term “dermoid cyst” specifically refers to MCTs that are predominantly composed of ectodermal derivatives.^22^ One hypothesis regarding their pathogenesis suggests that MCTs arise from parthenogenic embryogenesis within an ovum, which gives them their remarkable pluripotent capacity.^23^

Ultrasonography is considered the first-line imaging modality for the evaluation of adnexal masses. On US, mature cystic teratomas classically appear as heterogeneous lesions with hyperechoic foci and acoustic shadowing from calcifications, sebum, and hair. More specific sonographic findings include fat-fluid levels, a Rokitansky nodule, an iceberg sign, a dermoid mesh pattern, and a floating balls sign. Transvaginal US has been shown to have a sensitivity of 57.9% and specificity of 99.7% for detecting ovarian cystic teratomas, comparable accuracy to MRI. The heterogeneity, fat contents, and calcifications produce characteristic ultrasonographic features that aid in the diagnosis of mature cystic teratomas noninvasively.^22,24^

While most mature cystic teratomas remain benign, approximately 1.5%–2% may undergo malignant transformation. The most common malignancy arising in mature cystic teratomas is squamous cell carcinoma, typically developing from ectodermal elements.^25^ Interestingly, studies have found that the elevation of tumor markers like CA 125 and CA 19-9 does not correlate with malignant transformation to squamous cell carcinoma in these lesions. Therefore, while CA 125 and CA 19-9 can be elevated in benign mature cystic teratomas, these markers do not appear helpful for indicating malignant degeneration specifically into squamous cell carcinoma. Complete surgical excision and histopathological examination remain essential for assessing malignant features.^26^

Several retrospective studies have aimed to correlate CA 19-9 levels with specific clinical features of mature cystic teratomas. Some have found associations between higher CA 19-9 levels and larger tumor size, though statistical significance has been inconsistent.^27^ A positive correlation between CA 19-9, tumor size, and fat content has also been reported.^28^ However, other studies have failed to demonstrate a clear relationship between CA 19-9 elevation and meaningful parameters.^29^

Potential explanations for CA 19-9 elevation include leakage from a weakened cyst wall into circulation, especially in larger lesions.^18^ CA 19-9 rise may also relate to ovarian torsion and the degree of necrosis, possibly due to inflammatory changes from the ischemic reaction.^30^ Overall, CA 19-9 appears to be a sensitive marker for ovarian torsion and necrosis extent in mature cystic teratomas, for which prompt detection is clinically important.^19^ However, the precise etiology and clinical utility of CA 19-9 elevation require further elucidation.

In our prepubertal patient, the mature cystic teratoma was remarkably large at 13 × 11 cm (as measured postoperatively), and the CA 19-9 level was moderately elevated at 297 U/mL, though other tumor markers remained within normal limits. On laparotomy, the mass appeared grossly benign with a smooth surface and no adhesions or invasion into surrounding pelvic structures. Despite the large size, there was no evidence of torsion or rupture; however, the cyst wall was very thin and fragile with visibility of interior hair contents. Given the lack of malignant features and the patient’s young age, cystectomy alone with ovarian preservation was performed. Had malignant transformation been suspected intraoperatively, oophorectomy may have been warranted. Long-term follow-up for recurrence risk is planned, though the prognosis for this young patient is excellent after full resection of this benign lesion.

## 4. Conclusion

This case highlights an exceptionally large mature cystic teratoma in a pediatric patient, notable for an abnormally elevated CA 19-9 level. The tumor’s significant size and the unusual elevation of the tumor marker underscore the rarity of this presentation. Despite these atypical features, there were no complications such as torsion, rupture, or malignancy, suggesting a direct but unclear link between the teratoma and the rise in CA 19-9. A conservative, fertility-preserving surgical approach was judiciously chosen following a comprehensive preoperative evaluation. This report underscores the diverse manifestations of ovarian teratomas and reinforces the importance of meticulous clinical assessment and tailored management, particularly in pediatric patients.

## Conflict of Interest Statement

The authors declare that they have no competing interests.

## Ethics Approval and Consent to Participate

This case report involves a single patient who was under the care of Al-Hussein University Hospital, and the study was conducted in accordance with the ethical standards of the institution’s research committee. Written informed consent was obtained from the patient’s parent prior to inclusion in this study. Details that might disclose the identity of the patient have been omitted.

## Consent for Publication

Written informed consent was obtained from the patient’s parent for publication of the patient’s clinical details and accompanying images in this case report. A copy of the consent form is available for review by the Editor of this journal.

## Availability of Data and Materials

The datasets generated and/or analyzed during the current study are available from the corresponding author upon reasonable request.

## Authors’ Contributions

Both authors contributed equally to this work, involved in the preoperative assessment, surgical procedure, postoperative evaluation, writing of the initial manuscript, revising the manuscript, and read and approved the final manuscript.
